# Remittent Fever—Its Prompt Arrest and Effective Treatment

**Published:** 1887-04

**Authors:** John L. Jones

**Affiliations:** New Orleans, La.


### REMITTENT FEVER—ITS PROMPT ARREST AND EFFECTIVE TREATMENT.

BY JOHN L. JONES, M. D., NEW ORLEANS, LA.

To advance any new idea or discuss any theory in connection with the etiology or pathology of remittent fever is not here intended. My present purpose is simply to give a brief description of a general plan of treatment which has been attended by remarkably good results in my own practice and in my observation of the practice of others.

Crediting my confreres with as much therapeutical perspicuity and as keen powers for clinical observation as myself, I have seldom heretofore thought proper to trespass on the field of the journalistic writer, or attempt adding anything to our already heavily laden literature; but in this case my effort will, I trust, be considered commendable, inasmuch as this is one of the commonest diseases of our Southern country, and that there is no other disease in whose treatment more different practices are pursued or more varying results obtained.

Prof. Wood says: "There is perhaps no disease in which the resources of our profession are more happily displayed than in the worst forms of bilious fever. Fearfully fatal under neglect or mismanagement, they may, in the majority of cases, be conducted to a safe termination."

How often do we at the present day witness the sad illustration of this statement's truth. How often do we see two physicians practicing side by side, the practice of one being attended by death in a considerable proportion of the cases treated, whilst a death in the practice of the other, even from the most malignant type, is almost a rarity? Therefore I believe will go unquestioned the need for each to contribute his mite towards making this affection's treatment more efficacious, and to submit the results of his experience with it to the study and criticism of his professional brethren.

However, to give an intelligible account of the treatment, a short description of the disease itself or its more prominent symptoms will be necessary.

Remittent fever, bilious remittent fever, miasmatic fever with remissions, etc., is a malarial, continued fever, usually attended at regular periods with more or less well-marked remissions. These remissions are a more certain occurrence and a more definite characteristic of this affection than of any other febrile disease.

The fever usually begins with a sudden onset, either by a decided chill or rigors alternating with flashes of heat; it rises in the evening, is highest just before midnight, not so high after that hour, and the remission most occurs in the morning. Within the first twenty-four or forty-eight hours the pulse-beat may not be more than twenty or thirty above normal, but by the third or fourth day it may rise to one hundred and fifteen or one hundred and twenty. The tongue is broad, in the beginning moist, with a light white or yellowish white coating, perhaps red at the tip and edges, becoming, as the case advances, dry, browner or dark-colored, or chapped and fissured on the surface. During the febrile exacerbations the skin is usually harsh and dry, and the urinary secretion scanty and high-colored. Nervous and encephalic disorders are common, manifesting themselves by pain above one or both orbits, in the spinal column or the loins, or as nervousness, jactitation, delirium, etc. Portal engorgement, with constipated bowels and excess of bile production, is frequently present. The liver and spleen—the latter especially—often enlarged, there may be congestion of the stomach or of the intra-cranial and intra-spinal structures, the symptoms even sometimes indicating inflammation of some one or more of these parts. Albumen can very rarely be detected in the urine, at least in any specimen unmixed with blood. Hemorrhage, varying in amount and severity, may take place from the nose, lungs, stomach, intestines and kidneys—when from the four first the blood is either bright red and fluid or dark-colored and clotted, only very exceptionally presenting the grumous, coffee-ground appearance characteristic of the hemorrhage into the stomach in some severe forms or advanced stages of the yellow fever disease. Though there is a constant liability to the occurrence of hemorrhage from any of the organs named, it usually occurs, at least in any serious amount, as the result of sudden excitement, violent, nervous disturbance, inflammatory or congestive conditions, etc.

The types of the fever are quotidian, tertian and quartan (the first most often), sthenic and asthenic, mild and malignant, hemorrhagic, congestive, inflammatory, comatose, etc.

Very often sthenic, in the initial stage, it becomes asthenic when the case has a lengthened progress, so much so, perhaps, as to seem to merit the common, though misleading, name of typho-malarial.

The remissions are slow in their development and slow in their departure. However well marked in the beginning, if the fever continues some days unchecked, or runs into the asthenic type, they may become so indistinct and transient as to be scarcely at all perceptible, or only by a trifling abatement of some of the symptoms, such as the appearance of moisture on a previously dry skin, the allaying of the pains in different parts of the body, quieting of the nervousness, cessation of the nausea, a passage of more copious and clearer-colored urine, etc. This indistinctness of the remissions approaching almost to their entire absence is a not unfrequent characteristic of some of the more malignant forms of the fever.

*Treatment.*—However great may seem the indication in any case to allay the violence of the symptoms, or to diminish the height of the temperature, the physician's chief aim undoubtedly should always be the prompt and final arrest of the febrile exacerbations. For this purpose our sheet-anchor is quinine. *It ought to be given in one full and decided dose during the remission and then only.* No "penny wise and pound foolish" kind of economy ought to be tolerated in the administration of this medicine. My own practice is to give, on the first appearance of the remission, from 12 to 25 grains of bisulphate in solution, the alkaloid's most digestible form, in one or two draughts, to suit the stomach's tolerance. In administering it thus it will be found that a much smaller total amount will be required, and a quicker check put to the continuance of the fever than by any other method of using the drug.

I have no faith whatever in the use of quinine *in this disease* in any other way than as an anti-periodic. It has seemed to me that, given either in large or small doses during the absence of a remission, its tendency is to increase rather than diminish the fever, and to augment the gravity of the complications, particularly those of the head. Especially to be deprecated is the practice of administering small doses of the quinine, say from two to five grains in admixture with about the same quantity of calomel frequently repeated every hour or so during the height of the fever, I am led to believe by my observation of it in the hands of others. Calomel adulterated, as it generally is with a small quantity of the corrosive sublimate, is a powerful stimulant to the secretion of bile, which forms an insoluble compound with the quinine, or producing a purgative effect, as we might expect it to do in such doses, would, in either case, render the quinine inert, and the two together, by disturbing the bowels, would cause a reaction from the whole system.

Even other perceptible signs of an abatement were present. I have seen the quinine, given on the appearance of a slight moisture of the skin, very effective in bringing the fever under control. How this anti-periodic action is produced we can easily understand if we accept the view first advanced in 1878 by Prof. John B. Elliott, M. D., of the University of Louisiana, viz., that "fever results from a depression of that nervous center which controls the distribution of energy within the body, on account of which depression tissue building ceases, and the energy destined to perform that work passes into the lower form of heat." Knowing quinine to be a general nerve stimulant, we must suppose that it stimulates to proper activity this depressed centre at a time when it furnishes evidence that it is making an effort at the performance of its normal inhibiting function on the excess of heat generation. That its well-known anti-pyretic effect greatly assists its anti-periodic action is very probable, but that accurate clinical observation will corroborate the propriety of its use for its anti-pyretic effect alone I do not believe.

To show the efficiency of the method of using the quinine here advocated, I shall merely quote from a published letter of mine to Dr. Joseph Jones, at that time President of the Louisiana State Board of Health, dated Plaquemine parish, October 21st, 1880, while practicing in the central area of that fatal and malignant epidemic of remittent fever which that summer prevailed there. In my hands, when taken in the beginning, the fever has always been easily arrested by decided doses of quinine; thus treated I have seldom seen it last beyond four days, and in very few cases does it return after the first remission has occurred.

Altogether I have treated about one hundred and fifty or one hundred and seventy cases of this fever, and the total number of deaths of those seen and treated by me from the beginning is only two. In these two cases the fatal termination occurred several days after the fever had been arrested and the causes of death were entirely independent of the main disease.

(*To be Continued.*)

